# Relationship between the jaw-closing force and dietary form in older adults without occlusal support requiring nursing care

**DOI:** 10.1038/s41598-023-49059-4

**Published:** 2023-12-18

**Authors:** Rieko Moritoyo, Kazuharu Nakagawa, Kanako Yoshimi, Kohei Yamaguchi, Miki Ishii, Ryosuke Yanagida, Chizuru Namiki, Haruka Tohara

**Affiliations:** Division of Gerontology and Gerodontology, Department of Dysphagia Rehabilitation, Graduate School of Medical and Dental Sciences, Tokyo Medical and Dental University, 1-5-45 Yushima, Bunkyo-ku, Tokyo, 113-8510 Japan

**Keywords:** Health care, Medical research

## Abstract

In clinical practice, we encounter cases wherein older adults lacking occlusal support consume foods requiring mastication and adequate swallowing function. This study investigated the relationship between jaw-closing force (JCF) and dietary form in older adults without occlusal support requiring nursing care. This prospective cross-sectional study included 123 older adults requiring nursing care who lost their molar occlusal support and consumed food orally without dentures. JCF was defined as the force required for crushing food with the edentulous ridges or with the tooth and edentulous ridge while closing the mouth. Participants were classified into four groups based on the International Dysphagia Diet Standardization Initiative framework for recommended dietary forms. Basic information was collected, and tongue pressure and JCF were measured. Differences in JCF were analyzed using one-way analysis of variance, while factors related to dietary form were evaluated using ordinal logistic regression analysis. Significant differences in JCF were observed among the four groups. Factors such as the Barthel Index, tongue pressure, and JCF were dietary form-related. Our findings suggest that older adults requiring nursing care tend to have higher JCF when consuming meals requiring mastication. Therefore, JCF could serve as an index for determining appropriate dietary forms in this population.

## Introduction

As the global population ages, the number of older adults with suspected dysphagia is increasing. Individuals with suspected dysphagia are at risk of complications, such as malnutrition and respiratory problems due to aspiration^1,2^. Selecting an appropriate dietary form for residents with dysphagia is essential in nursing homes. To determine the appropriate dietary form, assessing not only swallowing but also masticatory function is important.

The masticatory function is closely associated with swallowing^3^. Moreover, occlusal support is related to occlusal force and masticatory function in older adults^4^. Generally, the greater the occlusal support in the molar region is, the better the masticatory function is^5^. Additionally, dental status strongly influences nutrition in older adults, on which reports indicated that having 20 or more remaining teeth was associated with good nutritional status^6,7^. On the other hand, occlusal force has been reported to be significantly related to diet and nutritional intake rather than number of teeth in older adults, which serves as an indicator of masticatory function^8^.

Many older adults requiring nursing care do not wear dentures due to underlying medical conditions that make visiting hospital difficult and, consequently, spend an extended period of their lives without occlusal support. Additionally, many individuals with reduced cognitive, oral, and manual motor functions may not wear dentures^9^. In clinical practice, there are cases wherein older adults who lack functional occlusal support do not use dentures but still consume food that requires chewing and display good bolus formation and swallowing function.

Various occlusal force measuring devices have been used for assessing masticatory function. One device involves placing a pressure-sensitive sheet over the entire dental arch and scanning it using a dedicated scanner to analyze occlusal pressure and balance^10^. Another device involves biting a tip with one of the molar teeth to quantify occlusal force^11^. These devices are only applicable to individuals with occlusal support, whereas it is impossible to measure occlusal force in individuals with edentulous or non-occlusal teeth.

In older adults with missing teeth, the tongue compensates for the masticatory function^12^: a relationship exists between maximum tongue pressure (TP) and dietary form^12,13^. However, whether the edentulous ridge could compensate for teeth function remains unclear. To date, no studies have quantified the ability of the edentulous ridges to crush food or examined its relationship to swallowing, which corresponds to bite force in older adults without occlusion.

Therefore, in collaboration with Murata Manufacturing Co. Ltd., we developed a device that could measure the ability of the edentulous ridges to crush food by thickening the sensor with a soft material. The jaw-closing force (JCF) meter comprises a body with a flexible sensor and protective film made from a porous material. The JCF could be measured by attaching the protective film to the flexible sensor and allowing the participant to bite the sensor part. The intra- and inter-rater reliability of the JCF meter in a pilot study of healthy young adults with occlusal support was significantly reproducible^14^.

In this study, we hypothesized that older adults requiring nursing care and having no molar occlusal support would be able to consume dietary forms that required mastication if they had a high JCF. We defined the force required to crush food with the edentulous ridges while closing the mouth as JCF and aimed to clarify its relationship with dietary form in older adults without occlusal support requiring nursing care.

## Pilot study

**Figure 1:**
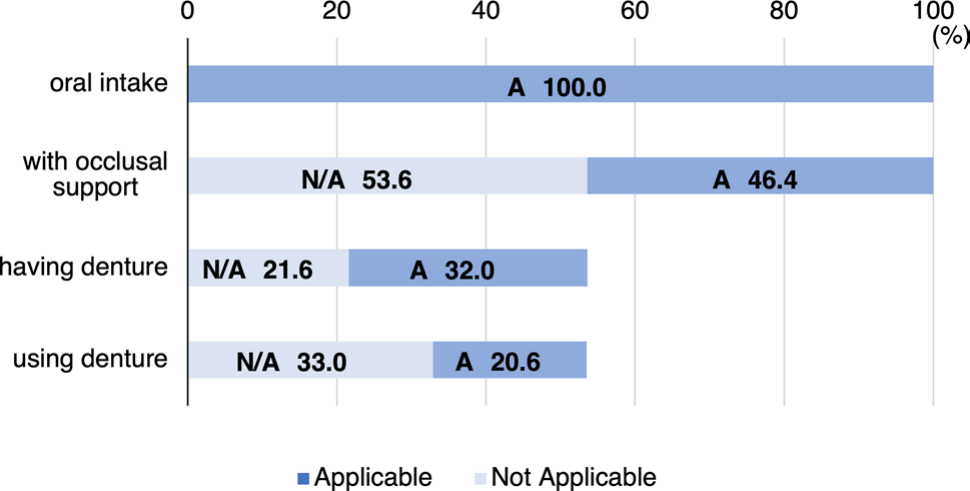
The proportion of older adults requiring nursing care who do not have molar occlusal support and do not use dentures for oral intake. Among 97 participants (100%), all consume food orally, and 52 (53.6%) have no molar occlusal support. Of the latter, 21 (21.6%) do not have dentures, and 32 (33.0%) of the older adults requiring nursing care consume food orally without using dentures whether they own them or not. *A* applicable, *N/A* not applicable.

As a preliminary survey, we determined the percentage of older adults without molar occlusal support requiring nursing care who could still consume food orally without dentures. The study was conducted including 97 residents in nursing homes who met the inclusion criteria until December 2021. Dental hygienists conducted interviews to assess nutritional intake and oral conditions. The survey items included whether the participants were able to consume food orally, had molar occlusal support, had dentures, or used them. The results of the survey revealed that among the 97 participants (27 males, 70 females, mean age 86.6 ± 7.6 years) who were included in the pilot study, all individuals consumed food orally. Among them, 52 individuals (53.6%) lacked molar occlusal support, whereas 45 participants (46.4%) did not. Among those without occlusal support, 21 individuals (21.6%) did not own dentures, whereas 31 (32.0%) owned dentures. Overall, 32 individuals (33.0%) did not use dentures. Notably, some individuals who owned dentures did not use them. Consequently, 63.5% of those without molar occlusion wherein denture use was recommended were eating without dentures (Fig. 1). The reasons for not using dentures included refusal of dental treatment, eating difficulty when wearing dentures, and inability to wear a denture due to discomfort or pain. A previous large study found that approximately 30% of community-dwelling older adults aged 65 years or older who have 19 or fewer teeth requiring dentures did not use fitting dentures^15^. In our pilot study, a higher percentage of older adults requiring nursing care living in facilities did not use dentures compared with community-dwelling older adults. Decline in activities of daily living (ADLs) and cognitive function were factors for the inability to use dentures despite presence of oral conditions that required dentures^9,16^. In addition, loss of dentures was a problem in nursing homes^17^. Moreover, caregivers faced barriers to managing oral health in older adults requiring nursing care^18^, which may also have made denture use more difficult. Therefore, based on the results obtained, we thought that assessing the appropriate dietary form for such older adults requiring nursing care without occlusal support who consumed food without dentures would be worthwhile.

## Methods

### Participants

**Figure 2:**
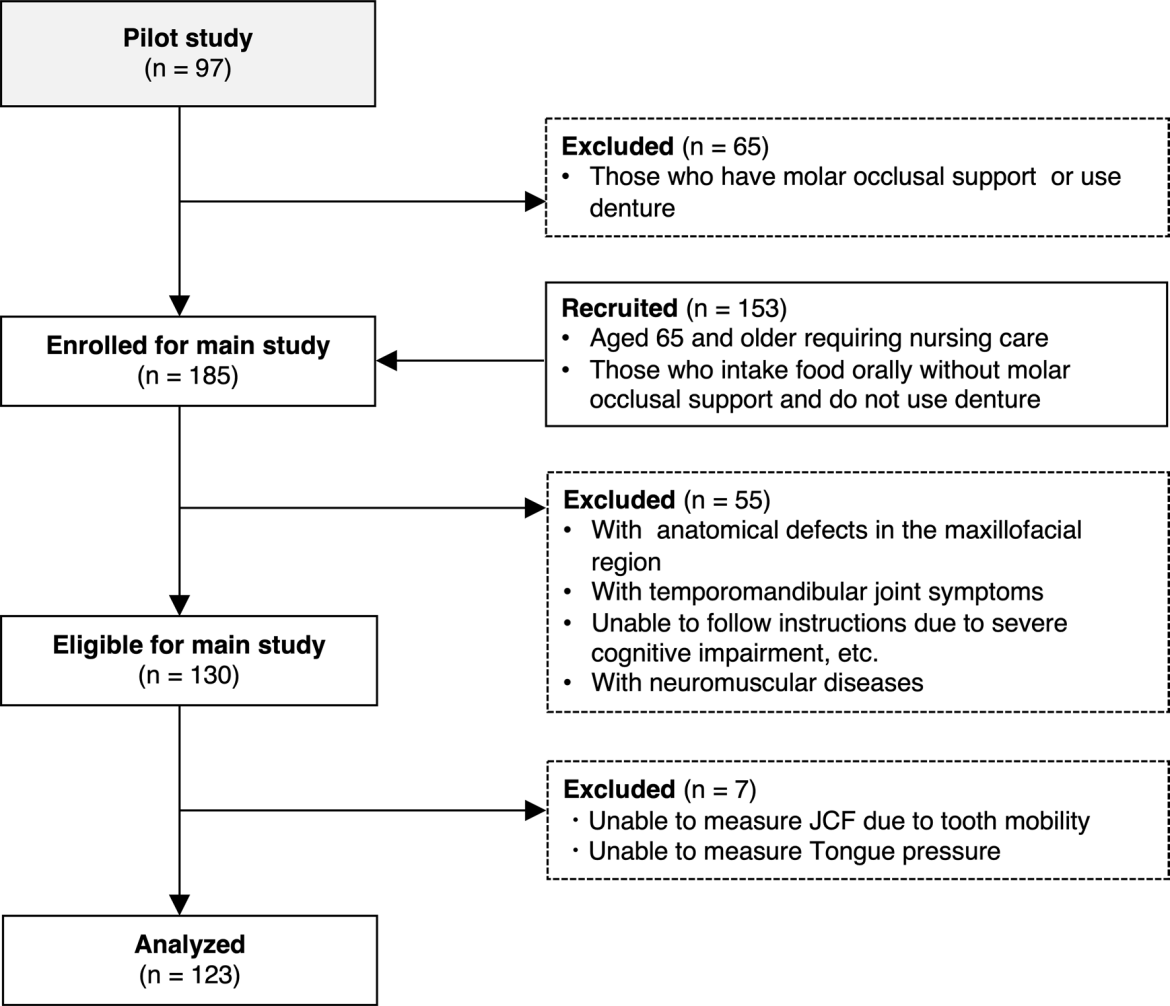
Flowchart of participant selection. Pilot study participants are also recruited into the main study, and participants in the main study are determined according to the arrows. Those who do not undergo JCF and TP measurements are additionally excluded.

This was a prospective cross-sectional study. From September 2021 to December 2022 and in October 2023, 185 older adults were recruited. The participants were among those who received home-visit medical care from the Department of Dysphagia Rehabilitation at Tokyo Medical and Dental University Hospital, resided in nursing homes, or were inpatients in a rehabilitation hospital and scheduled for discharge during the study period. The inclusion criteria were individuals: (1) aged 65 years or older and certified as requiring nursing care under Japan’s long-term care insurance system^19,20^, (2) without molar occlusal support (Eichner classification B4 or higher), (3) ate without dentures on a daily basis (4) whose primary method of nutritional intake was oral, and (5) provided informed consent to participate in the study. The exclusion criteria were individuals with: (1) anatomical defects in the maxillofacial region, (2) temporomandibular joint symptoms, and (3) Clinical Dementia Rating^21^ score 3 (severe) or with score 2 or less who were unable to follow the study procedures, (4) and neuromuscular diseases (Fig. 2). The study protocol was approved by the Institutional Review Board of TMDU (Approval ID: No. D2020-024). All participants provided informed consent prior to enrollment in the study.

### Data collection

We collected data on age, sex, height, weight, dentition, medical history, and activity status. We calculated body mass index (BMI) from height and weight measurements and assessed the participants’ occlusal status based on their dentition. We collected the Charlson Comorbidity Index (CCI)^22^ from the medical history and Barthel Index (BI) scores from the activity status. Previous studies have validated the reliability of the BI^23^.

### Measurements

#### Eichner classification and occlusal condition

The participant's oral status was recorded by a dentist. The degree of tooth mobility and presence of residual roots were checked. Occlusal status was evaluated using the Eichner classification^24^. The occlusal support regions were classified into four areas: the right and left premolars and the right and left molars. The Eichner classification was categorized into three main groups: Eichner A (occlusal support in all four areas), Eichner B (occlusal support in one, two, or three areas or only in the anterior teeth), and Eichner C (no occlusal support). The participants in this study were classified as Eichner B4, who had no occlusal support in the molars and had it only in the anterior teeth, and Eichner C. We recorded Eichner B4 or Eichner C.

The occlusal condition was classified into two groups of molar regions where the JCF was measured: "ridge-to-ridge" for occlusion by the upper and lower edentulous jaw or "ridge-to-teeth" for occlusion by either the upper or lower edentulous jaw and opposing remaining teeth. The area with residual roots was defined as an edentulous ridge.

#### Tongue pressure

TP was measured using a TP measurement device (JMS Co. Ltd., Hiroshima, Japan). The participants were instructed to sit down, hold a balloon in their mouth, and close their lips while holding a plastic probe at the level of their upper and lower central incisors or the corresponding part of their edentulous ridge. The dentist maintained the probe in the correct position. The participants were instructed to press the balloon against their hard palate with their tongue and maintain maximum pressure for 7 s. The measurements were performed three times, after which the average values were recorded^25,26^. Those who could not maintain TP for 7 s or those who could not undergo TP measurement were excluded from the study.

#### The JCF

**Figure 3:**
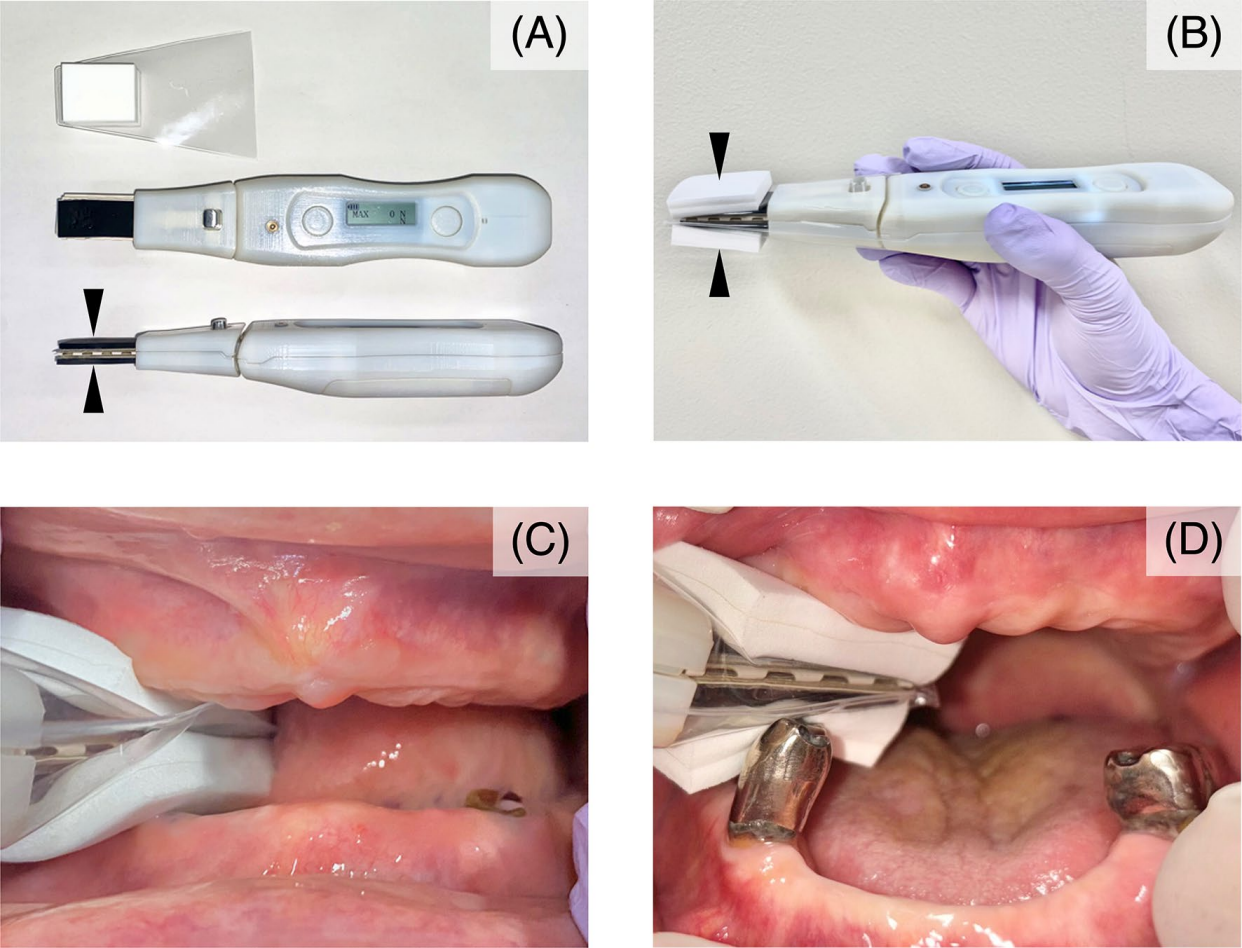
Jaw-closing force meter. (**A**) The top is the protective film, and the bottom is the jaw-closing force meter. The arrow indicates a flexible sensor, 8 mm thick. In the shaft’s center is a display showing the jaw-closing force (N). (**B**) Arrows indicate that the protective film is attached to the flexible sensor, with a total thickness of 20 mm, and the device is held as shown. The porous material is attached to the outside of the protective film. (**C**) Occlusal condition is ridge-to-ridge; the sensor's center is inserted into the molar area of the edentulous ridge. (**D**) Occlusal condition is ridge-to-teeth; the sensor's center is inserted into the remaining molar.

The JCF was measured using a prototype JCF meter (Fig. 1A) developed in collaboration with Murata Manufacturing Co., Ltd. It comprises a central unit with a thick flexible sensor mounted on its end and a protective film used to avoid interference of remaining teeth during occlusion (Fig. 3A). For measuring the JCF, the 12 mm thick protective film was applied to the 8 mm thick flexible sensor at the tip of the JCF meter (total thickness, 20 mm) (Fig. 3B), and the center of the sensor was inserted into the molar area of the edentulous ridge (Fig. 3C, D). While sitting, participants were instructed to bite down with maximum force on the soft sensor covered by the protective film. When pressure was applied to the sensor, the force was displayed in Newtons on the central display of the main unit. If there was a mobile tooth with mobility of class II or higher according to Miller's classification^27^ at the measurement site, the JCF was measured avoiding that area. If the JCF meter and mobile tooth were in contact, the measurement was stopped. Those who could not undergo JCF measurement were excluded from the study. The JCF was measured three times on each side. Thereafter, the mean value was calculated for the left and right sides, with the higher value taken as the highest measured value in any given position.

#### Recommended dietary form

The recommended dietary form for participants was classified by dental professional personnel knowledgeable in dysphagia rehabilitation based on observation of the participants’ eating habits and other assessments, using the International Dysphagia Diet Standardization Initiative (IDDSI) framework. Dietary forms were classified into levels from 3 to 7 based on the methods described on the official website of the IDDSI (https://www.iddsi.org)—level 3: liquidized foods that do not require oral processing or chewing and could be swallowed directly; level 4: pureed foods that do not require biting or chewing; level 5: minced and moist foods that require minimal chewing; level 6: soft, bite-sized foods that require chewing; level 7: regular foods that require adequate chewing ability. An endoscopic evaluation of swallowing was performed in those who had pharyngeal dysphagia and required evaluation of food mass formation, including the presence of aspiration^28^. Finally, level 3 and level 4, which do not require chewing, were grouped, resulting in a total of four groups.

### Data analysis

To determine the differences among the four recommended dietary form groups in participant characteristics, continuous variables such as age, BMI, CCI, BI, Eichner classification, TP, and JCF were analyzed using the Shapiro–Wilk test to confirm normal distribution. These variables were then analyzed using one-way analysis of variance and the Kruskal–Wallis test. Categorical variables, such as sex, Eichner classification, and occlusal condition were analyzed using chi-square and Fisher's exact tests. The Bonferroni test was used for post-hoc analyses. The receiver operating characteristic (ROC) curve was used to determine the JCF cut-off point for distinguishing between the different diet groups. The area under the curve (AUC) was obtained from the ROC curve, and the point closest to the coordinate point of the upper left corner (sensitivity = 1; specificity = 1) was calculated as the cut-off value. The analysis was performed using levels 5 and 6 as the boundaries between the food forms generally eaten by people without teeth and those likely to be judged as inedible. Spearman's rank correlation coefficient was used to analyze the correlation between JCF and each item.

To adjust for confounding factors, ordinal logistic regression analysis was performed using recommended dietary form as the objective variable and age, sex, BMI, CCI, BI, TP, and JCF as explanatory variables.

The sample size was 103 participants with a power of 0.8, α = 0.05, an effect size of 0.15 (medium), and the number of explanatory variables was 7. The significance level was set at 5% or less. The Statistical Package for Social Sciences (SPSS) version 28.0.1.1 (IBM Japan, Tokyo, Japan) was used for all statistical analyses.

### Ethics approval

All procedures performed in studies involving human participants were in accordance with the ethical standards of the institutional and/or national research committee and with the 1964 Helsinki Declaration and its later amendments or comparable ethical standards. The study protocol was approved by the Ethical Committee of Faculty of Dentistry, Tokyo Medical and Dental University (Approval ID: No. D2020-024) and registered at the University Hospital Medical Information Network Center in Japan (UMIN number: UMIN000044020).

### Consent

Informed consent was obtained from all individual participants included in this study or their legal guardians.

## Results

### Comparison of participant characteristics among the four groups

In total, data of 123 participants (38 males and 85 females) were included in the analysis. The mean age was 85.8 ± 7.1 years. The participants were divided into four groups based on the recommended dietary form: level 3–4 (n = 23, 18.7%), level 5 (n = 22, 17.9%), level 6 (n = 47 38.2%), and level 7 (n = 31, 25.2%). The normality of continuous variables was confirmed: age and BMI were found to be normally distributed, whereas CCI, BI, TP, and JCF were not. Participant characteristics are presented in Table 1. There were significant differences in age, sex, BMI, CCI, BI, and JCF among the groups (*p* < 0.05, Table 1). Twenty-four participants underwent video endoscopy.

**Table 1:** Comparison of participant characteristics among the four groups (n = 123).

Characteristics	Level 3–4 (n = 23)	Level 5 (n = 22)	Level 6 (n = 47)	Level 7 (n = 31)	*p*-value
Age, mean ± SD	88.7 ± 5.5	86.0 ± 7.3	85.2 ± 8.1	82.6 ± 7.8	0.013*^†^
Sex, N (%)					
Male	3 (13.0)	4 (18.2)	18 (38.3)	13 (41.9)	0.044*^§^
Female	20 (87.0)	18 (81.8)	29 (61.7)	18 (58.1)	
BMI (kg/m^2^), mean ± SD	17.8 ± 2.8	18.8 ± 4.1	18.8 ± 3.0	21.7 ± 3.8	< 0.001*^†^
CCI, median (IQR)	2.0 (2.0–2.0)	2.0 (2.0–2.0)	2.0 (0.0–2.0)	2.0 (0.0–2.0)	0.034*^‡^
BI, median (IQR)	5.0 (0.0–25.0)	15.0 (3.8–31.3)	40.0 (10.0–65.0)	65.0 (45.0–80.0)	< 0.001*^‡^
Eichner classification					
B4	1 (4.3)	3 (13.6)	7 (14.9)	8 (2.6)	0.199^∮^
C	22 (95.7)	19 (86.4)	40 (85.1)	23 (7.4)	
TP (kPa), median (IQR)	6.6 (4.3–13.7)	10.1 (5.4–15.8)	17.9 (10.5–22.7)	27.6 (18.9–34.8)	< 0.001*^‡^
JCF (N), median (IQR)	33.3 (30.0–53.3)	46.7 (39.2–50.8)	60.0 (43.3–96.7)	90.0 (73.3–123.3)	< 0.001*^‡^
Occlusal condition, N (%)					
Ridge-to-ridge	19 (82.6)	18 (81.8)	30 (63.8)	23 (74.2)	0.263^§^
Ridge-to-teeth	4 (17.4)	4 (18.2)	17 (36.2)	8 (25.8)	

We recorded whether the occlusal condition of the JCF measurement site was ridge-to-ridge or ridge-to-teeth. Based on the IDDSI, recommended dietary forms were classified from level 3 to level 7, with level 3 and level 4 in the same group: level 3, liquidized; no oral processing or chewing required—can be swallowed directly; level 4, pureed; no biting or chewing is required; level 5, minced and moist; minimal chewing is required; level 6, soft and bite-sized; chewing is required; level 7, regular; ability to bite hard or soft foods and chew them for a long time.

*SD* standard deviation, *IQR* interquartile range, *BMI* body mass index, *CCI* Charlson Comorbidity Index, *BI* Barthel Index, *TP* tongue pressure, *JCF* jaw-closing force, *OC* occlusal condition.

*p < 0.05.

^†^One Way-ANOVA test.

^‡^Kruskal–Wallis test.

^§^Chi-square test.

^∮^Fisher's exact tests.

### Comparison of the JCF among the four groups

**Figure 4:**
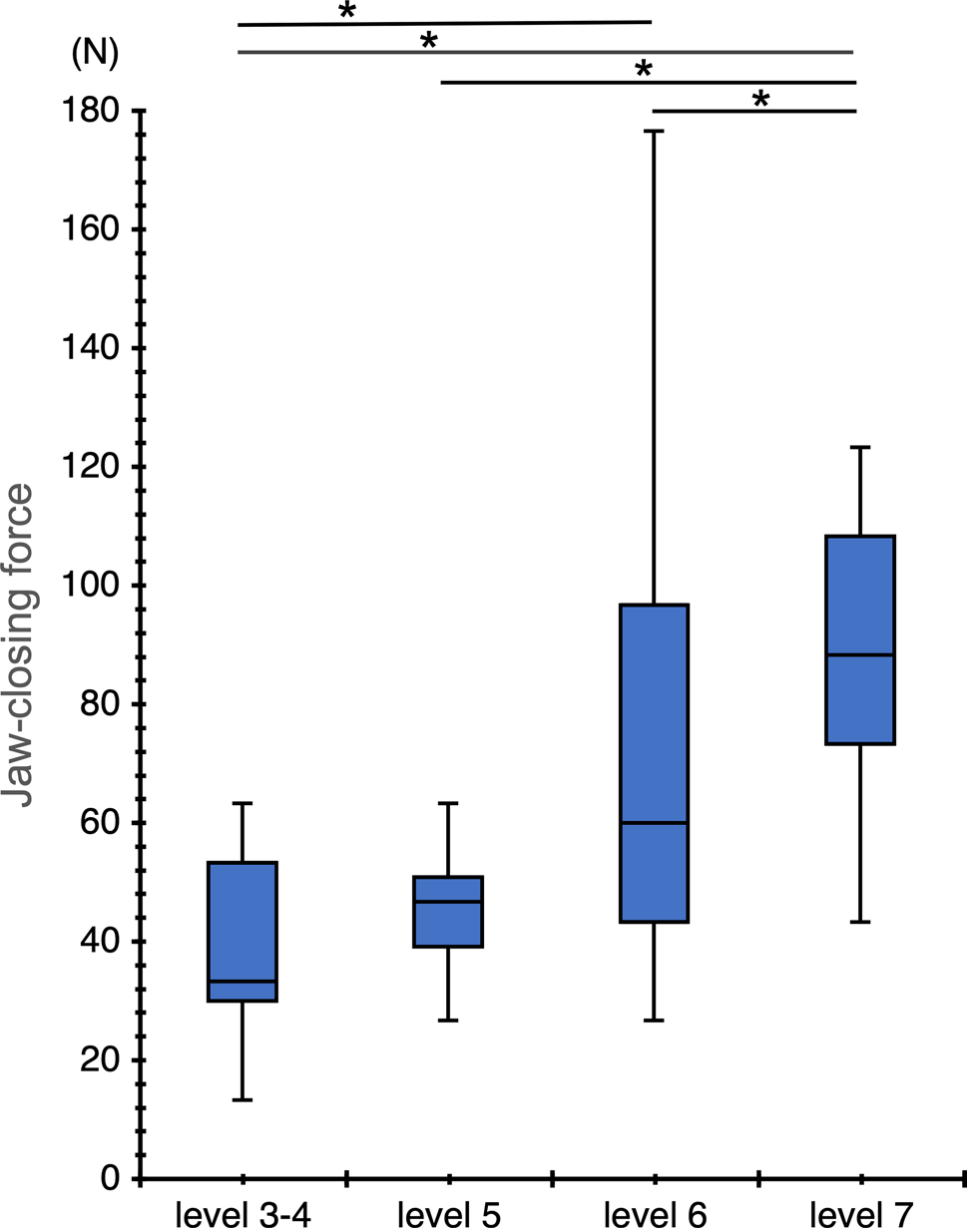
Comparison of jaw-closing force. The graph shows the analysis results regarding significant differences in jaw-closing force between the four groups divided according to IDDSI. The data are analyzed using the Kruskal–Wallis test and a post hoc test using the Bonferroni correction. These graphs show the median value and interquartile range. *p < 0.05.

The median JCF was 33.3 (interquartile range [IQR], 30.0–53.3) N for levels 3–4, 46.7 (IQR, 39.2–50.8) N for level 5, 60.0 (IQR, 43.3–96.7) N for level 6, and 90.0 (IQR, 73.3–123.3) N for level 7. A significant difference was observed between the groups. Post-hoc tests were performed for JCF. The measurement of JCF highlighted significant differences between level 3–4 and level 6; level 3–4 and level 7; level 5 and level 7; and level 6 and level 7 (Fig. 4).

Regarding the ROC analysis, the cut-off value for JCF between level 5 or below and level 6 or above was 61.2 (N), with a sensitivity of 0.628 and a specificity of 0.911. The AUC was 0.836 (*p* < 0.001).

### Correlation between the JCF and each item

The JCF values showed a strong correlation with BI (ρ = 0.368), TP (ρ = 0.449), and the recommended dietary form (ρ = 0.515). They demonstrated a weak correlation with age (ρ = -0.260), sex (ρ = -0187), and BMI (ρ = 0.262) and no correlation with the CCI, Eichner classification, and occlusal condition (Table 2).

**Table 2:** Correlation coefficients for each item (n = 123).

	Age	Sex	BMI	CCI	BI	Eichner	TP	JCF	OC	IDDSI
Age	1									
Sex	0.233**	1								
BMI	−0.129*	−0.051	1							
CCI	0.020	−0.045	−0.060	1						
BI	−0.185**	−0.170*	0.215**	−0.179*	1					
Eichner	0.118	−0.042	−0.047	0.184*	−0.114	1				
TP	−0.095	−0.097	0.215**	−0.159*	0.411**	−0.097	1			
JCF	−0.260**	−0.187*	0.262**	−0.124	0.368**	−0.115	0.449**	1		
OC	−0.120	−0.032	0.034	−0.045	0.165*	−0.300**	−0.030	0.146	1	
IDDSI	−0.188**	−0.221**	0.262**	−0.210**	0.480**	−0.173*	0.457**	0.515**	0.088	1

We recorded whether the occlusal condition of the JCF measurement site was ridge-to-ridge or ridge-to-teeth. Based on the IDDSI, recommended dietary forms were classified from level 3 to level 7, with level 3 and level 4 in the same group: level 3, liquidized; no oral processing or chewing required—can be swallowed directly; level 4, pureed; no biting or chewing is required; level 5, minced and moist; minimal chewing is required; level 6, soft and bite-sized; chewing is required; level 7, regular; ability to bite hard or soft foods and chew them for a long time. Spearman rank correlation coefficient, *p < 0.05, **p < 0.001.

*BMI* body mass index, *CCI* Charlson Comorbidity Index, *BI* Barthel Index, *Eichner* Eichner classification, *TP* tongue pressure, *JCF* jaw-closing force, *OC* occlusal condition.

### Factors affecting dietary form

Significant explanatory factors for dietary form were BI (B = 0.021, 95% CI for B = 0.006 to 0.036, *p* = 0.006), TP (B = 0.073, 95% CI for B = 0.027 to 0.120, *p* = 0.002), and JCF (B = 0.016, 95% CI for B = 0.003 to 0.030, *p* = 0.016). There was no significant correlation between the dietary form and age, sex, BMI, or CCI (Table 3).

**Table 3:** Results of ordinal logistic regression analysis of the dietary form (n = 123).

		B	95% CI for B	*p*-value
Age		−0.009	(−0.062, 0.044)	0.735
Sex		−0.413	(−1.237, 0.411)	0.326
BMI		0.07	(−0.041, 0.180)	0.216
CCI		−0.152	(−0.440, 0.136)	0.301
BI		0.021	(0.006, 0.036)	0.006*
TP		0.073	(0.027, 0.120)	0.002*
JCF		0.016	(0.003, 0.030)	0.016*
Model: *p* < 0.001; Nagelkerke’s R^2^-square: 0.539				

*B* partial regression coefficient, *95% CI* 95% confidence interval, *BMI* body mass index, *CCI* Charlson Comorbidity Index, *BI* Barthel Index, *TP* tongue pressure, *JCF* jaw-closing force.

*p < 0.05.

## Discussion

### JCF meter

In this study, we developed a new prototype JCF meter that could measure the ability to crush food with the edentulous ridges or with the tooth and edentulous ridge while closing the mouth. The JCF meter could overcome the main drawback of the existing pressure-sensitive film methods, which cannot be applied to individuals with edentulous jaws or those with lost occlusal support in the molars. While devising the JCF meter prototype, we tested the probe in older adults with various inter-arch distances. As there was a concern that the JCF might be lower when alveolar bone resorption was severe, we tested different protective film thicknesses (resulting in a total probe thickness of 10, 14, 16, or 20 mm) in several Eichner classification B4 and C occlusal conditions in order to determine the appropriate thickness of the protective film to be attached to the JCF meter's 8 mm thick flexible sensor. The results showed that a total thickness of 20 mm allowed the JCF to be measured without interference from the anterior teeth. Therefore, we set the probe thickness to 20 mm to ensure that sufficient force could be exerted. In this way, the force to crush an object between the edentulous ridges or between the edentulous ridge and opposing teeth with the mouth closed could be measured, regardless of the resorption level of the alveolar bone.

### Relationship between JCF and recommended dietary form

The JCF differed significantly among the four recommended dietary forms and was significantly higher in those who received a diet requiring mastication. Using ROC curve analysis, the cut-off value for levels 5 and 6 was 61.2 (N) with a sensitivity of 0.628, and an exceptionally high specificity of 0.911. Older adults without molar occlusal support requiring nursing care are often offered foods that do not require mastication, and approximately 90% of these individuals may be able to consume soft, bite-sized foods if the JCF is higher than approximately 60 (N). The JCF may be used as a screening parameter for determining the appropriate dietary form for older adults.

### Correlation between the JCF and each item

First, JCF showed a strong positive correlation with the IDDSI level. On the other hand, Eichner classification and occlusal status did not correlate with JCF.

Since JCF correlated with occlusal force in those with occlusal support^14^, the number of teeth could affect JCF in those with ridge-to-teeth occlusion. However, in older adults without occlusal support requiring nursing care, JCF may not be necessarily related to Eichner classification and the number of remaining teeth.

Second, JCF was strongly correlated with TP. Although the muscles that work when JCF is exerted have not been clarified, the masseter and jaw-closing muscles are involved in lifting the mandible and chewing. Therefore, JCF might be related to muscular forces that reflect oral functions such as TP.

In a previous study of healthy older adults, there was a correlation between JCF and age, but not between JCF and BMI^18^. In the present study, JCF was correlated with age, BMI, and BI, an indicator of activity level. Thus, nutritional status and activity level as well as TP and occlusal force were found to be associated with JCF in older adults requiring nursing care.

### Factors affecting dietary form

The significant factors associated with dietary form were BI, TP, and JCF, which were not significantly associated with age, sex, BMI, or CCI.

The tongue is essential for masticatory swallowing^29–31^. It carries ingested food to the molars and pushes the chewed food mass backward and into the hypopharynx during swallowing^32^. It has already been recognized that TP could influence dietary form^13^. However, JCF also has a significant effect on dietary form, as the crushing of the food by the JCF and its transfer by the tongue motor function in a coordinated manner is thought to result in proper food bolus formation. Although the number of teeth and occlusal support strongly influence masticatory ability^33^, this is the first study to clarify the relationship between the crushing ability by edentulous ridges and diet, independent of the number of teeth and occlusal support.

However, the presence of teeth may have had a negligible effect on JCF. As listed in Table 1, some of the participants had a remaining molar in either the upper and lower jaws, and JCF was measured with the edentulous ridge and teeth. The residual tooth may have exerted more force due to the shorter distance between the measured sites. The occlusal condition of the participants in this study was a mix of ridge-to-ridge and ridge-to-teeth, but their proportions were comparable between the recommended dietary forms. Thus, the influence of the remaining teeth on JCF and recommended dietary forms was minimally adjusted.

Denture use is an essential factor in determining dietary form^34^. When selecting a dietary form for older adults requiring long-term care who are unable to wear dentures due out of necessity, more careful attention should be paid to them than to those who use dentures. When a detailed swallowing examination is not available, the JCF screening test could be beneficial in determining the appropriate dietary form. If JCF was added as a new indicator for selecting dietary forms for those who lack occlusal support or cannot use dentures, the dietary life of older adults requiring nursing care could be enriched.

### Limitations

JCF can be used as a measure to evaluate the ability to crush food but not the ability to grind or pulverize it. A portion of the participants was administered the Saku-Saku Test^35^, which assesses the ability to grind, but we could not recruit sufficient participants for analysis.

This study focused on participants who primarily consumed nutrition orally and essentially excluded those with pharyngeal dysphagia or acute-phase swallowing disorders. In future studies, it would be necessary to consider such patients by incorporating other screening tests along with the current assessments.

This is a cross-sectional study evaluating the relationship between JCF and diet form that could be consumed, and the reference values for JCF as an indicator of oral function might not be clear. If future studies could provide an indicator of JCF, it would be more useful for safe food intake by older adults who require nursing care.

## Conclusions

The JCF was higher in the older adults requiring nursing care without molar occlusal support who consumed a diet requiring mastication. The JCF meter used in this study could evaluate mastication and food bolus formation abilities, regardless of dental status. Clinically, JCF may help determine the optimal dietary form for older adults without molar occlusal support requiring nursing care.

## Data Availability

The datasets generated during and/or analyzed during the current study are available from the corresponding author on reasonable request.
